# Myopericytoma of the Right Middle Finger: A Case Report of an Uncommon Perivascular Neoplasm

**DOI:** 10.7759/cureus.61938

**Published:** 2024-06-08

**Authors:** Babatope L Awosusi, Omar M Attia, John Nwadiokwu

**Affiliations:** 1 Pathology and Laboratory Medicine, King Khalid Hospital, Al Majma'ah, SAU; 2 Plastic Surgery, King Khalid Hospital, Al Majma'ah, SAU; 3 Anatomic Pathology, Babcock University Teaching Hospital, Ilishan-Remo, NGA

**Keywords:** myopericytoma, pericytic, perivascular neoplasm, soft tissue neoplasm, finger swelling

## Abstract

This report describes the case of a 29-year-old male who presented with painless swelling on the volar aspect of his right middle finger. The initial clinical impression was consistent with an epidermal inclusion cyst. A plain radiograph of the lesion revealed a circumscribed superficial nodular soft tissue mass confined to the dermis of the affected finger. Following surgical excision and subsequent histopathologic examination, the lesion was ultimately identified as a myopericytoma (MPC). The occurrence of MPCs in the finger is uncommon; thus, a high level of suspicion is required to consider it as one of the differential diagnoses for painless nodules in this anatomical location. Surgery serves as the primary method for treatment, and histopathologic evaluation plays a crucial role in confirming the diagnosis and ruling out malignancy.

## Introduction

Myopericytomas (MPCs) are rare lesions that were initially described by Granter et al. in 1998 as benign neoplasms originating from pericytes [1]. They were designated as soft tissue tumors by the World Health Organization (WHO) in 2022 [2]. Typically, MPCs affect the dermis and subcutaneous tissues of the lower extremities [3,4].

MPCs consist of myoid-like cells that exhibit an ovoid shape with smooth muscle differentiation. These cells are arranged in a concentric perivascular pattern and typically express actin [3].

The occurrence of MPCs in the hand is uncommon. In a systematic review and case report by Morzycki et al., only 22 cases of hand and wrist MPCs were reported, with just six previous cases located in the finger [5]. A more recent review by Lim et al. in 2022 described an additional two cases of finger MPCs [6].

In this article, we present the case of a 29-year-old male with a painless swelling on the volar aspect of his right middle finger, which was ultimately diagnosed as an MPC.

## Case presentation

A 29-year-old male presented with painless swelling on the volar aspect of the right middle finger, which had been present for six months. There was a positive history of slow, progressive increase in size, with no other associated symptoms or history of trauma. Additionally, there was no significant past medical history.

During the physical examination, a small swelling measuring 1.5x1cm was observed on the volar aspect of the right middle finger. The swelling was nodular, partly mobile, firm, and non-tender. No swelling was observed in other parts of the body, and systemic examination was unremarkable. The initial clinical impression was an inclusion/implantation cyst.

A plain radiograph of the lesion showed a circumscribed superficial nodular soft tissue lesion limited to the dermis of the right middle finger. Other laboratory investigations conducted were unremarkable.

The patient underwent surgical excision of the lesion under local anesthesia, and the sample was sent for histopathologic evaluation. The sample received at surgical grossing was a small nodule measuring 1.5x1.5 cm in dimension, with cut sections showing firm tan-white surfaces.

**Figure 1:**
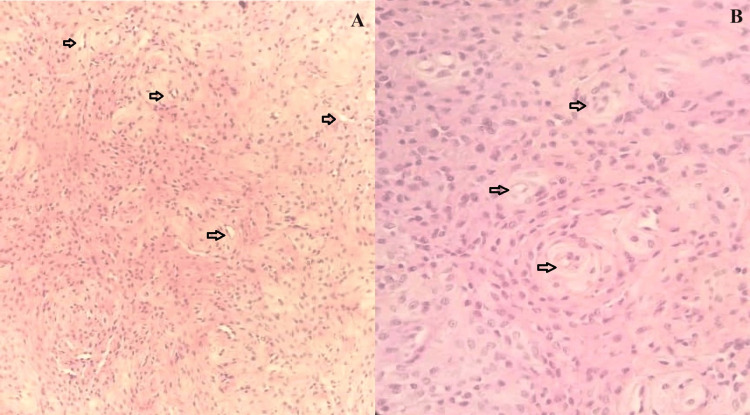
Photomicrograph of the MPC showing spindle-shaped myoid cells with perivascular arrangements (black arrows). A: Stained with hematoxylin and eosin; x100 magnification. B: Stained with hematoxylin and eosin; x200 magnification. MPC: Myopericytoma

**Figure 2:**
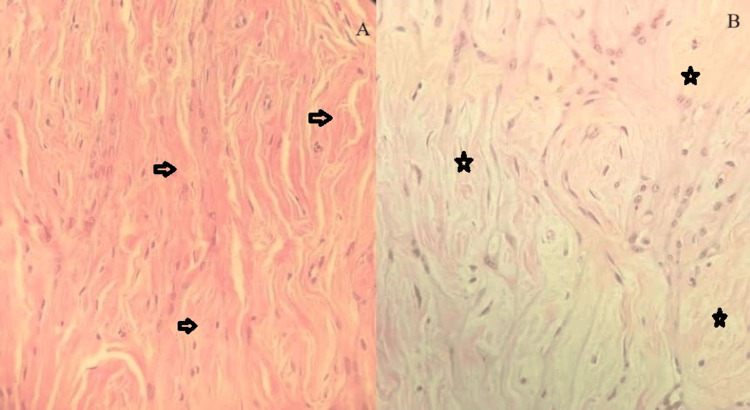
Photomicrograph of the MPC showing collagenous (black arrows) and hypocellular areas (black stars). A: Stained with hematoxylin and eosin; x100 magnification. B: Stained with hematoxylin and eosin; x200 magnification. MPC: Myopericytoma

Microscopic examination revealed a nodular, well-circumscribed mesenchymal neoplasm composed of proliferations of bland, oval to spindle-shaped myoid cells with multilayered, concentric growth around numerous small thick-walled blood vessels (see Figures 1A, 1B). The lesion also showed variable cellularity, ranging from cellular to hypocellular and collagenous (see Figures 2A, 2B).

No focus on necrosis or mitotic activity was observed. Based on the morphological characteristics of the tumor cells, a definitive diagnosis of an MPC was made. The patient remained stable post-operatively with no adverse outcomes.

## Discussion

MPCs predominantly affect males and can occur in all age groups [4]. This epidemiology is consistent with the present case; the lesion was observed in a young adult male.

Most patients with MPCs typically present with a slow-growing, solitary, and non-painful nodular soft tissue swelling [4]. While MPCs commonly occur in the extremities, they have also been reported in other parts of the body, including the neck, stomach, and kidneys [4]. In this case, our patient presented with an MPC of the right middle finger, which is quite uncommon. After a thorough literature search of the PubMed and Google Scholar databases, only 10 previous cases of finger MPCs were found (refer to Table 1).

**Table 1:** Characteristics of cases of finger myopericytomas available in the literature.

Author	Year of publication	Sex	Age	Location	Associated symptom	Presence of recurrence
Mentzel et al. [7]	2006	Male	55 years	Finger (subcutis)	NA	NA
Mentzel et al. [7]	2006	Female	13 years	Finger (dermis)	NA	NA
Mentzel et al. [7]	2006	Male	52 years	Finger (subcutis)	NA	NA
Mentzel et al. [7]	2006	Male	62 years	Finger (subcutis)	NA	NA
Sadahira et al. [8]	2015	Male	70 years	Periungual (subcutis)	NA	No
Mahapatra et al. [9]	2015	Female	59 years	Digital artery (intravascular)	Pain	No
Camp et al. [10]	2017	Female	46 years	Base of fourth and fifth fingers (dermis)	Difficulties in daily activities	No
Morzycki et al. [5]	2017	Male	33 years	Finger (subcutis)	Asymptomatic	No
Kumar et al. [11]	2021	Male	83 years	Proximal phalanx of index finger	Pain	
Lim et al. [6]	2022	Female	66 years	Finger (dermis)	Asymptomatic	No
Present case		Male	29 years	Finger (dermis)	Asymptomatic	No

Though the etiopathogenesis of MPCs is still largely unknown, recent studies have revealed some genetic abnormalities, such as platelet-derived growth factor receptor-beta (PDGFRB) mutations, NOTCH receptor 3 (NOTCH3) mutations, serum response factor, and NOTCH rearrangements as being associated with various pericytic tumors. Among these genetic abnormalities, PDGFRB somatic mutations are associated with sporadic myopericytomas [12]. However, some authors have proposed trauma [13] and AIDS as possible triggers [14]. There was no history of trauma or immunodeficiency in this case. The lesion was reported to have started spontaneously and was initially thought to be an epidermal inclusion cyst.

Histopathologically, the MPC is characterized by the proliferation of round-to-ovoid spindle-shaped cells with eosinophilic cytoplasm arranged in a perivascular concentric pattern [3,4,15]. Some cases may exhibit blood vessels arranged in hemangiopericytoma-like patterns [15]. In this case, histopathologic evaluation after surgical excision showed morphologic features consistent with MPC.

In challenging cases, immunohistochemistry can be used to confirm the diagnosis and rule out close differentials. The MPC typically tests positive for SMA, h-caldesmon, and vimentin immunohistochemical antibodies, while it generally tests negative for CD34, CD68, S100, and desmin [15].

Surgical excision is the primary treatment [4,16]. Following excision, histopathological evaluation is necessary to confirm the diagnosis and rule out malignancy [15]. The MPC typically follows a benign clinical course, but the recurrence rate may range from 10% to 20% if surgical excision is incomplete [15]. A few cases of malignant MPCs have been reported in the literature [4,17]. No focus of atypia or malignant change was observed in this case, and the patient remained free of any recurrence after 12 months of follow-up at the surgical outpatient clinic.

## Conclusions

An MPC is a rare benign soft tissue neoplasm with a slow, indolent course. Its occurrence in the finger is uncommon. A high index of suspicion is required to consider it as one of the differential diagnoses of painless nodules of the finger. Surgery is essential for diagnosis, and histopathologic evaluation is crucial to confirm and rule out malignancy.
